# Timeline of SARS-CoV-2 Spread in Italy: Results from an Independent Serological Retesting

**DOI:** 10.3390/v14010061

**Published:** 2021-12-30

**Authors:** Emanuele Montomoli, Giovanni Apolone, Alessandro Manenti, Mattia Boeri, Paola Suatoni, Federica Sabia, Alfonso Marchianò, Valentina Bollati, Ugo Pastorino, Gabriella Sozzi

**Affiliations:** 1 Department of Molecular and Developmental Medicine, University of Siena, 53100 Siena, Italy; emanuele.montomoli@unisi.it; 2 VisMederi S.r.l., 53200 Siena, Italy; alessandro.manenti@vismederiresearch.com; 3 Scientific Direction, Fondazione IRCCS Istituto Nazionale Tumori, 20133 Milan, Italy; Giovanni.apolone@istitutotumori.mi.it; 4 VisMederi Research S.r.l., 53100 Siena, Italy; 5 Department of Research, Fondazione IRCCS Istituto Nazionale Tumori, 20133 Milan, Italy; mattia.boeri@istitutotumori.mi.it; 6 Department of Surgery, Fondazione IRCCS Istituto Nazionale Tumori, 20133 Milan, Italy; paola.suatoni@istitutotumori.mi.it (P.S.); federica.sabia@istitutotumori.mi.it (F.S.); 7 Department of Radiology, Fondazione IRCCS Istituto Nazionale Tumori, 20133 Milan, Italy; alfonso.marchiano@istitutotumori.mi.it; 8 EPIGET-Epidemiology, Epigenetics and Toxicology Lab., University of Milan, 20100 Milan, Italy; valentina.bollati@unimi.it

**Keywords:** SARS-CoV-2, antibodies, serologic

## Abstract

The massive emergence of COVID-19 cases in the first phase of pandemic within an extremely short period of time suggest that an undetected earlier circulation of SARS-CoV-2 might have occurred. Given the importance of this evidence, an independent evaluation was recommended by the World Health Organization (WHO) to test a subset of samples selected on the level of positivity in ELISA assays (positive, low positive, negative) detected in our previous study of prepandemic samples collected in Italy. SARS-CoV-2 antibodies were blindly retested by two independent centers in 29 blood samples collected in the prepandemic period in Italy, 29 samples collected one year before and 11 COVID-19 control samples. The methodologies used included IgG-RBD/IgM-RBD ELISA assays, a qualitative micro-neutralization CPE-based assay, a multiplex IgG protein array, an ELISA IgM kit (Wantai), and a plaque-reduction neutralization test. The results suggest the presence of SARS-CoV-2 antibodies in some samples collected in the prepandemic period, with the oldest samples found to be positive for IgM by both laboratories collected on 10 October 2019 (Lombardy), 11 November 2019 (Lombardy) and 5 February 2020 (Lazio), the latter with neutralizing antibodies. The detection of IgM and/or IgG binding and neutralizing antibodies was strongly dependent on the different serological assays and thresholds employed, and they were not detected in control samples collected one year before. These findings, although gathered in a small and selected set of samples, highlight the importance of harmonizing serological assays for testing the spread of the SARS-CoV-2 virus and may contribute to a better understanding of future virus dynamics.

## 1. Introduction

The timeline of the first COVID-19 cases remains an unanswered question [1,2]. The first declared case of COVID-19 worldwide was dated on 8 December 2019, in Wuhan, China. In Europe, although local transmission was only identified in the second half of February in most countries, there is accumulated evidence that SARS-CoV-2 circulated before this and possibly before the first cases identified in Wuhan.

This hypothesis is supported by several published studies including environmental waste water testing [3,4] as well as seroprevalence and molecular retrospective analyses on clinical samples of asymptomatic and symptomatic subjects. Positive SARS-CoV-2 RT-PCR results were reported in France in a patient with pneumonia on 27 December 2019 [5] and in both a child with suspected measles and a woman with extended skin dermatosis in November 2019, in Milan, Italy [6,7].

Large seroprevalence studies in the USA and in Europe support an earlier than expected circulation of the virus. Retrospective SARS-CoV-2 serological testing of 7389 routine blood donations collected in nine U.S. states from 13 December 2019–17 January 2020 suggested that the virus was present as early as 13–16 December 2019 [8].

Using serum samples routinely collected in 9144 adults from a French general population-based cohort, 353 participants with a positive anti-SARS-CoV-2 IgG test were identified. Notably, 13 participants with positive ELISA-S had been sampled between 5 November 2019 and 30 January 2020 and were confirmed by neutralizing antibodies testing. In these positive subjects, the authors identified symptoms, a history of possible exposure or specific events compatible with early SARS-CoV-2 infection [9].

The Italian Ministry of Health accomplished a large SARS-CoV-2 seroprevalence study in a representative sample of 64,660 subjects collected between 25 May and 15 July 2020. A global prevalence rate of 2.5% was reported, with a peak in the Lombardy region (7.5%) and in particular in the Province of Bergamo (24%) (www.salute.gov.it (accessed on 15 July 2021)). According to these numbers, the true number of Italians who had been in contact with the virus would be approximately 1.5 million, many of which were asymptomatic, an estimate, which is almost five times higher than the official figures, reported at that time, suggesting that SARS-CoV-2 was circulating below the surface.

We investigated the presence of SARS-CoV-2 Receptor-Binding Domain (RBD)-specific antibodies in blood samples of 959 asymptomatic individuals enrolled in the SMILE prospective lung cancer screening trial (clinicaltrials.gov ID: NCT03654105) between September 2019 and March 2020 and across all the Italian regions. SARS-CoV-2 IgM/IgG RBD-specific antibodies were detected in 111 of 959 (11.6%) subjects, starting from September 2019 (14%), with a cluster of positive cases (>30%) on the 2nd week of February 2020 and the highest number (53.2%) in Lombardy [10]. The publication of our report generated a lively debate on the possibility that the virus circulated months earlier in Italy without surveillance programs that were able to identify any signs of its presence. To validate these findings, we were encouraged to blindly retest a selection of our samples and paired controls in an external WHO-affiliated laboratory (Erasmus Medical Center, Rotterdam) by using different serological assays. Here, we report the results of this cross-validation study.

## 2. Materials and Methods

The sample series included 29 plasma samples collected in the SMILE trial (clinicaltrials.gov ID: NCT03654105) between 23 July 2019 and 17 February 2020 [10] and 29 plasma samples from another lung cancer screening cohort (bioMILD, clinicaltrials.gov ID: NCT02247453) collected between 14 July 2018 and 23 February 2019, matched by date of collection, sex, age and smoking habits. The Institutional Review Board and Ethics Committee of Fondazione IRCCS Istituto Nazionale dei Tumori of Milan approved the study. All eligible subjects provided written informed consent. Nine samples from asymptomatic and symptomatic convalescent COVID-19 patients with a positive molecular (RT-PCR) swab collected from the National Institute for Biological Standard and Control (NIBSC), the University Siena and the University of Milan, as well as two commercial (BioIVT, West Sussex, UK) negative control samples, were also included.

VisMederi, an independent laboratory which is part of the CEPI consortium (https://cepi.net (accessed on 15 July 2021)), used proprietary IgG-RBD/IgM-RBD ELISA assays and a qualitative Micro-Neutralization CPE-based assay (MN) with the aim of increasing the sensitivity of the test [11,12]. The assays were qualified and validated following the guidelines issued by the International Council for Harmonization of Technical Requirements for Pharmaceuticals for Human Use (ICH) (www.ich.org (accessed on 15 July 2021)). A cut-off value of each plate was obtained by multiplying the “BLANK” optical density (OD) signal three times, which is derived from six micro-wells containing sample diluents and a secondary HRP-antibody but no analyte [12]. Using Vismederi standards, the criteria for indicating the presence of SARS-CoV-2 specific antibodies was RBD-specific IgM and/or IgG above the cut-off value, with or without the presence of neutralizing antibodies [12].

Erasmus tested the samples with a multiplex IgG protein array including three antigens: S1, ecto and nucleocapsid protein (NP). According to Erasmus standards, a criteria of IgG triple antigen positives, with confirmation of neutralization assay using a Plaque Reduction Neutralization Test (PRNT), were needed before scoring a sample as positive. For the purpose of IgM detection, Erasmus adopted the ELISA IgM commercial kit Wantai (Wantai, Beijing, China) using cut-off, as suggested by the manufacturer [13,14]. The specificity of both the VisMederi and Erasmus assays was validated across the most common HCoV sera [12,13].

For both the VisMederi and Erasmus assays, the IgM results were calculated by relating each specimen OD value to the respective cut-off value of the plate. Results were thus expressed as and OD ratio and considered positive when >1. For correlation analysis of nonparametric data, the Spearman rho coeficent (r) with a respective 95% confidence interval and a two-tailed *p*-value was calculated. To compare data distributions, *p*-values were calculated using the Mann–Whitney test. The GraphPad Prism software (version 5.2) was adopted for the purpose of analysis and graphic design.

## 3. Results

### 3.1. Retesting of Lung Cancer Screening Participants

According to the Vismederi results of the 29 SMILE samples, seven cases were positive for IgG, sixteen cases were classified as positive (*n* = 13) or borderline (*n* = 3) for IgM, of which six samples were also positive in qualitative microneutralization assay and six cases were negative (Table 1). According to the Erasmus Medical Center, none of the SMILE series showed IgG triple antigen positivity (three samples had S1 or S1+NP positivity only), two samples were positive and one was in the grey zone for IgM detection using Wantai cut-offs (Table 1). Out of the twelve tested samples for the PRNT assay, one IgM positive sample also had neutralizing antibodies (Table 1).

**Figure 1:**
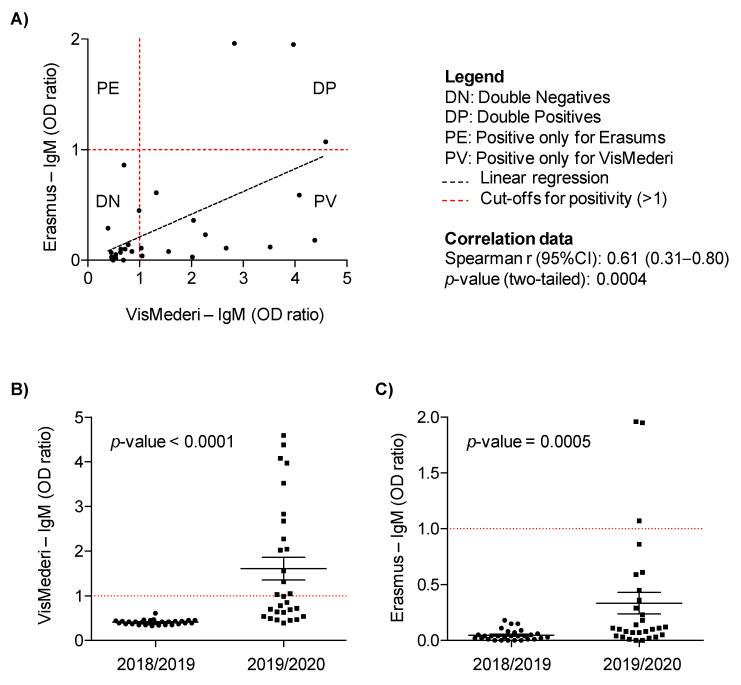
Comparison of IgM data between VisMederi and Erasmus. (**A**) The scatter plot shows the correlation between IgM values (OD ratios) obtained in the two laboratories, considering the 29 selected samples collected from asymptomatic subjects enrolled in the SMILE lung cancer screening trial between July 2019 and February 2020. The dot plots show the distribution of IgM values (OD ratio) obtained by (**B**) VisMederi and (**C**) Erasmus in 29 samples of the SMILE screening trial enrolled in 2019/2020 and 29 matched lung cancer screening volunteers enrolled in the same months of 2018/2019. Horizontal black bars indicate mean value ± standard error. Mann–Whitney *p*-values are reported.

In nine additional samples, classified as IgM positives by VisMederi, a signal was also detected by Erasmus, but below the threshold level of the Wantai test (Figure 1A). These differences, especially in IgM outcomes, would seem to derive from a different setting of the cut-off values used to attribute positivity or negativity to anti SARS-CoV-2 antibodies. In fact, the ELISA cut-off of VisMederi was established internally through a blind study of symptomatic and asymptomatic subjects positive to molecular swab [15]. On the other hand, the Wantai ELISA commercial kit uses a different cut-off set up for diagnostic purposes in symptomatic patients. Despite this, a significant correlation among IgM values (VisMederi vs. Erasmus) was observed (Spearman r = 0.6130; *p* = 0.0004), when OD ratios were considered as continuous variables (Figure 1A).

To better assess the specificity of assays, 29 plasma samples collected between July 2018 and February 2019 from subjects matched to those of the SMILE study and supposed to be negative were also included. Indeed, all these samples resulted in negatives using both VisMederi and Erasmus IgG and IgM assays. By plotting IgM values recorded by both centers, a significantly higher distribution of values was observed in samples collected in 2019–2020 versus control samples collected one year before in 2018–2019: *p* < 0.0001 and *p* = 0.0005 by VisMederi and Erasmus, respectively (Figure 1B,C).

### 3.2. Analysis of COVID-19 Control Samples

In order to have a better insight into the performances of assays and the concordance between the two laboratories, eleven positive convalescent patients (three symptomatic and six asymptomatic) and negatives controls (*n* = 2) were included in the study (Table 2). RBD specific IgG were detected by VisMederi in all nine positive symptomatic and asymptomatic COVID-19 subjects, whereas only the three symptomatic convalescents samples were classified as triple positive in a multiple IgG protein array by Erasmus. None of the analyzed samples from asymptomatic convalescents tested positive in neutralization assays whereas the three samples from symptomatic convalescents were shown to have neutralizing activity by both the Vismederi and Erasmus (titers: 1280, 40, 40 respectively) laboratories.

## 4. Discussion

Overall, the results of this blind retesting of a selected set of samples indicate the presence of SARS-CoV-2 antibodies in some SMILE samples collected in the prepandemic period. The oldest samples found positive for IgM by both laboratories were collected on 10 October 2019 (Lombardy), 11 November 2019 (Lombardy) and 5 February 2020 (Lazio), the latter with neutralizing antibodies. Two additional samples collected on 17 December 2019 (Campania) and 28 January 2020 (Lombardy) tested as IgG positive by VisMederi and positive for IgG S1 and IgG S1+NP by Erasmus. Additional IgM positive cases could have been detected also by Erasmus by lowering the cut-off of the commercial IgM assay. The older among these putative additional IgM positive samples was collected on 3 September 2019 in the Veneto region, one of the first and mostly severely affected COVID-19 regions.

It is worth noting that for samples from asymptomatic SARS-CoV-2 infected subjects, where the antibody concentration is known to be very low and are rarely able to develop neutralizing antibodies [16,17,18], the agreement among the different tests used in the two laboratories is poor, highlighting the issue of sensitivity in commercial serological tests. Conversely, samples from symptomatic COVID-19 convalescents tested positive for IgG and showed neutralizing activity by both the laboratories. If it is true that high specificity is an important parameter in SARS-CoV-2 seroprevalence studies, even sensitivity is an equally important parameter that should be considered when testing asymptomatic infected subjects.

Erasmus standards dictate that triple IgG antigen positive criteria with neutralization test confirmation are used in order to classify a sample as positive. This is based on in house validation data for surveys of pre-outbreak sera in order to set a high specificity value for studies in which it is important to be able to have conclusive results. Indeed, the percentage of triple positive sera increases starting at 7 days post infection, indicating lower sensitivity in recently (<7 days) infected subjects. Accordingly, by using this approach, only symptomatic COVID-19 control patients were identified while asymptomatic controls and SMILE subjects did not reach the triple antigens cut-off. In contrast, IgM antibodies and neutralizing activity were found in a SMILE sample collected on 5 February, two weeks before the first officially declared COVID-19 Italian patient.

Lombardy was the first Italian region affected by the COVID-19 outbreak, with a death rate almost six times higher than in the rest of the country. A recent large phylogenetic study on 346 SARS-CoV-2 patients in Lombardy allowed for the identification of seven SARS-CoV-2 lineages and the presence of local transmission clusters within three of them, suggesting that the virus was circulating undetected for some time before first detection and confirming the central role of Lombardy in the SARS-CoV-2 epidemic [19]. Similar conclusions were reached in a previous report in a smaller number of sequenced samples [20]. In contrast, no evidence of SARS-CoV-2 viral RNA was found in 1581 respiratory samples collected in the framework of influenza surveillance between October 2019 and February 2020 in Lombardy [21]. Nonetheless, the presence of neutralizing antibodies in five blood donors collected at the beginning of February in the Lodi Red Zone, where the outbreak started, was reported by the same authors [22].

Beyond serological studies and computational genomic phylogenetic analyses, the isolation of the SARS-CoV-2 viral genome in both a respiratory swab of a symptomatic child recovered for suspected measles and the skin biopsy of a symptomatic woman with positive IgG Sars-CoV-2 antibodies later on, both in November 2019, in Milan, strongly supports the early undetected circulation of the virus [6,7]. In a recent preprint, Amendola et al. extended the molecular and immunological study to 44 prepandemic samples with morbilliform rashes collected between August 2019 and February 2020 in Lombardy [23]. They reported the finding of 11/44 (25%) positive subjects with first positivity in a urine sample collected on 12 September 2019, with four of them also showing anti-Sars-CoV-2 antibodies with IgM as the most frequent antibody class. None of the 100 samples collected from August 2018–July 2019 tested positive for any molecular or serological assay. Sequencing of prepandemic cases identified strains with at least six mutations away from the inferred Sars-CoV-2 progenitor strain, suggesting the existence of a Sars-CoV-2 progenitor already in late June to late August 2019. Interestingly, their conclusions are in keeping with the present serological findings.

A detailed analysis of the COVID-19 epidemic in January 2020, the month preceding its detection in Italy and in the successive weeks until the outbreak, was recently provided [24]. Retrospective epidemiological investigations identified 527 laboratory-confirmed cases with symptom onset before the detection of the first COVID-19 diagnosed patient (20 February 2020) and suggested that SARS-CoV-2 was already circulating in at least 222 out of 1506 (14.7%) municipalities, with sustained transmission across all the Lombardy provinces. The authors highlighted that the high transmissibility of the infection and the widespread silent transmission of Sars-CoV-2 between January and mid-February 2020 caused the rapid increase in COVID-19 patients during a period when restrictive control measures had not yet been implemented due to a lack of awareness regarding the circulation of COVID-19.

These findings do not at all suggest that the virus originated in Italy, but they endorse the idea that the virus was likely spreading in China before the first known cases and that could have been circulated by travelers given direct the connections between China and European and US countries, particularly the Northern West and East Italian regions, which are among the most industrialized and connected areas of Italy. Evidence used to support this hypothesis comes from a comparative genomic analysis of more than 175,000 genomes, which delineated 22 distinct SARS-CoV-2 haplogroups with a broad geographic distribution within China, pointing towards an early emergence and widespread cryptic circulation of the virus well before its isolation in January 2020 [25]. Recently, Kumar et al. reconstructed the mutational history of SARS-CoV-2 using a so called ‘mutation order approach’ (MOA) [26]. From their analysis of more than 174,000 genomes, major mutational fingerprints revealed that it is useful to identify and track the spatiotemporal evolution of novel coronavirus. The progenitor genome identified differed from that of the first coronaviruses sampled in China by three variants, implying that none of the earliest patients represents the index case or gave rise to all human infections. However, multiple coronavirus infections in China, the USA and Europe harbored the progenitor genetic fingerprint in January 2020 and later, suggesting that the progenitor was spreading worldwide months before.

A recently published letter by Petti et al. summarizes existing evidence that corroborates the infectious disease epidemiology principle of pathogen circulation prior to the recognized outbreak [27]. The eventuality of an early SARS-CoV-2 circulation already relatively sustained in Europe and America is not so astonishing, as SARS-CoV-2 is mainly a respiratory pathogen. Therefore, a novel unknown respiratory virus responsible for severe pneumonia like SARS-CoV-2 could circulate undetected for months or years, be responsible for many deaths, and even become a pandemic, before peculiar characteristics of the disease are noticed that allow for its identification.

An alternative hypothesis of circulation of a genetically different virus with reduced transmissibility and/or virulence cannot be excluded. This could also explain why we observed a low degree of neutralization for plasma samples from 2019. The results of this study might suggest that SARS-CoV-2 circulated unrecognized until it mutated and gained the capacity to cause severe disease and pandemic capacity.

We acknowledge some limitations in this re-testing study related to the small sample size, the highly specific cohort of screening participants (heavy smokers ≥ 30 pack years and ≥ 55 years old), possibly not representative of the general population, and the intrinsic experimental variability of the immunoenzymatic assays employed in the different laboratories. We cannot exclude that other confounding conditions, such as preexisting immunity against other agents, might have contributed to the SARS-CoV-2 positivity in our assays. Nonetheless, cross reactivity towards the most common HCoVs was ruled out. Furthermore, a large series of pre-outbreak plasma samples of the BioMILD study collected during the same time frame (September 2018–February 2019) were recently tested with the Vismederi IgG/IgM assays and only a few (<2%) showed a low reactivity, supporting the accuracy of the methodology employed in our study.

As pointed out in the recent WHO report [28] and in other commentaries [1,2], studies from different countries suggest that SARS-CoV-2 was growing undetected for some time before the first diagnosed case in Wuhan. The suggestive evidence of our previous published study [10] and the conflicting results from the current retesting exercise on a small and selected subset of samples do not allow us to accept or discard this hypothesis. Indeed, the findings of these studies are only partially confirmed due to the heterogeneity of methods utilized and the risk of non-specific signals in serological assays. Despite this, the report underlines the importance of investigating these potential early events in order to solve the still unanswered questions about the origin and timing of the current pandemic and to better understand future virus circulation dynamics.

## Figures and Tables

**Table 1:** Serological results of the SMILE series 2019–2020.

Date of Collection	Italian Region	IgG		IgM		Neutralization Assay	
		VisMederi	Erasmus	VisMederi	Erasmus	MN VisMederi	PRNT Erasmus
23 July 2019	N/A	Neg	Neg	Neg	Neg	N/A	N/A
24 July 2019	N/A	Neg	Neg	Neg	Neg	N/A	N/A
3 September 2019	Lombardy	Neg	Neg	Neg	Neg	N/A	N/A
3 September 2019	Veneto	Neg	Neg	Pos (RBD)	Neg	Neg	N/A
5 September 2019	Liguria	Neg	Neg	Borderline	Neg	Neg	N/A
10 September 2019	N/A	Neg	Neg	Neg	Neg	N/A	N/A
12 September 2019	Lombardy	Neg	Neg	Neg	Neg	N/A	N/A
17 September 2019	N/A	Neg	Neg	Neg	Neg	N/A	N/A
2 October 2019	Piedmont	Pos (RBD)	Neg	Neg	Neg	Neg	Neg
7 October 2019	Valle’d’Aosta	Neg	Neg	Pos (RBD)	Neg	Pos	N/A
7 October 2019	Liguria	Pos (RBD)	Neg	Neg	Neg	Neg	Neg
7 October 2019	Lombardy	Neg	Neg	Pos (RBD)	Neg	Pos	N/A
8 October 2019	Lombardy	Neg	Neg	Pos (RBD)	Neg	Pos	N/A
10 October 2019	Lombardy	Pos (RBD)	Neg	Neg	Neg	Neg	Neg
10 October 2019	Lombardy	Neg	Neg	Pos (RBD)	Pos (RBD)	Neg	Neg
15 October 2019	Lombardy	Neg	Neg	Pos (RBD)	Neg	Neg	N/A
15 October 2019	N/A	Neg	Neg	Neg	Neg	N/A	Neg
21 October 2019	Tuscany	Neg	Neg	Pos (RBD)	Neg	Pos	N/A
22 October 2019	Lombardy	Neg	Neg	Neg	Neg	N/A	N/A
7 November 2019	Lombardy	Neg	Neg	Borderline	Neg	Pos	N/A
11 November 2019	Lombardy	Neg	Pos (S1)	Pos (RBD)	Borderline	Neg	Neg
26 November 2019	Lazio	Pos (RBD)	Neg	Neg	Neg	Neg	Neg
13 December 2019	Lombardy	Pos (RBD)	Neg	Borderline	Neg	Neg	Neg
17 December 2019	Campania	Pos (RBD)	Pos (S1)	Pos (RBD)	Neg	Neg	Neg
18 December 2019	Liguria	Neg	Neg	Pos (RBD)	Neg	Neg	N/A
28 January 2020	Lombardy	Pos (RBD)	Pos (S1,NP)	Neg	Neg	Neg	Neg
5 February 2020	Lazio	Neg	Neg	Pos (RBD)	Pos (RBD)	Pos	Pos
12 February 2020	Lombardy	Neg	Neg	Pos (RBD)	Neg	Neg	Neg
17 February 2020	Piedmont	Neg	Neg	Pos (RBD)	Neg	Neg	N/A

Neg: Negative; Pos: Positive; N/A: Not available; RBD: Receptor-Binding Domain; NP: Nucleocapsin Protein.

**Table 2:** Serological results in negative controls, convalescent symptomatic and asymptomatic COVID-19 patients.

Sample	Center of Collection	IgG		IgM		Neutralization	
		VisMederi	Erasmus	VisMederi	Erasmus	MN VisMederi	PRNT Erasmus
Negative control	NIBSC	Neg	Neg	Neg	Neg	Neg	N/A
Negative control	NIBSC	Neg	Neg	Neg	Neg	Neg	N/A
Convalescent symptomatic	NIBSC	Pos (RBD)	Pos (S1, ecto, NP)	Neg	N/A	Pos	Pos
Convalescent symptomatic	BioIVT	Pos (RBD)	Pos (S1, ecto, NP)	Pos (RBD)	Pos (RBD)	Pos	Pos
Convalescent symptomatic	BioIVT	Pos (RBD)	Pos (S1, ecto, NP)	Pos (RBD)	Neg	Pos	Pos
Convalescent asymptomatic	UNISI	Pos (RBD)	Neg	Neg	Neg	Neg	N/A
Convalescent asymptomatic	UNIMI	Pos (RBD)	Neg	Neg	N/A	Neg	Neg
Convalescent asymptomatic	UNIMI	Pos (RBD)	Neg	Neg	Neg	Neg	N/A
Convalescent asymptomatic	UNIMI	Pos (RBD)	Neg	Neg	Neg	Neg	N/A
Convalescent asymptomatic	UNIMI	Pos (RBD)	Neg	Neg	Neg	Neg	N/A
Convalescent asymptomatic	UNIMI	Pos (RBD)	Neg	Neg	N/A	Neg	Neg

Neg: Negative; Pos: Positive; N/A: Not available; RBD: Receptor-Binding Domain; NP: Nucle-ocapsin Protein.

## Data Availability

Data are available upon request to the corresponding authors.
